# Public Health Legislation in 1921

**Published:** 1922-02

**Authors:** 


### PUBLIC HEALTH LEGISLATION IN 1921.

### A GENERAL REVIEW.

Although the past year was not marked by any striking changes in the law of public health, some important Acts found their way to the Statute Book.

The removal of Sanatorium benefit from the list of benefits given under the Health Insurance Acts made it necessary to pass the Tuberculosis Act, 1921. County and County Borough Councils were empowered by the first Insurance Act to provide treatment for tuberculosis, and were encouraged to do so by Exchequer grants administered by the Local Government Board. They are now under a statutory obligation to make adequate arrangements for the purpose; but any Council which was already providing treatment under a scheme approved by the Board (or its successor, the Ministry of Health) is to be deemed to have made adequate arrangements so long as the scheme continues in operation. Since the great majority of the Councils had schemes in force, the practical change effected by the Act is not great; but statutory recognition of the fact that the treatment of tuberculosis is a public obligation constitutes an important step in advance. The Act also empowers the Councils to make arrangements for the after-care of persons who have been under treatment, and effects other minor amendments of the law.

The Dentists Act, 1921, prohibits the practice of dentistry by unqualified persons, and sets up a Dental Board, to which is entrusted the duty of keeping the dentists' register, and to which are transferred the existing powers of the General Medical Council under the Dentists Act, 1878. The persons entitled to be admitted to the register are defined by the Act. These include, subject to the fulfilment of conditions, certain classes of persons who were practising dentistry in the British Isles at the commencement of the Act, but might not be otherwise qualified. Provision is also made prohibiting the carrying on of the business of dentistry by dental companies save under the conditions specified in the Act. A right of appeal to the High Court is given against a removal from the register or a refusal to register. The Act has been warmly welcomed by the dental profession, and though many will wish that it had gone even further than it does, its results are likely to prove beneficial in the extreme in the course of a few years.

Two small Health Insurance Acts became law. The first makes certain adjustments in the finance of the insurance scheme, and also reduces the size of Insurance Committees whose work has diminished with the disappearance of sanatorium benefit. The second enables persons who are in danger of passing out of insurance by reason of a prolonged period of unemployment to retain (subject to certain conditions) their right to benefit.

The Housing Act, 1921, extended the power to give subsidies to private builders for another eighteen months, but immediately after the Act had become law the Government announced that the subsidy would be wholly discontinued. The Act also amended the Housing Acts in some minor details, and authorised the Public Works Loan Commissioners, with Treasury sanction, to advance money to authorised associations for the development of garden cities.

The Public Health (Officers) Act, 1921, gives security of tenure to Medical Officers of Health, and also to Sanitary Inspectors, by which name all Inspectors of Nuisances are henceforward to be known.

Among other Acts, we may note the Coroners Act, 1921, which increases the remuneration of Coroners; the Water Undertakings (Modification of Charges) Act, 1921, which empowers the Ministry of Health to allow water undertakers to increase their water charges to meet increased expenses; the Health Resorts and Watering Places, Act, 1921, which authorises local authorities to expend certain moneys not exceeding the equivalent of a rate of one penny in the pound in advocating the attractions of their district; and the Licensing Act, 1921, which removes some of the war-time restrictions on the sale of intoxicating liquor, though happily it does not restore pre-war conditions. The Safeguarding of Industries Act may, it is feared, increase the price of certain classes of scientific instruments and glass.

Lastly, the Education Act, 1921, has consolidated all the existing statute-law on the subject of education, including the law relating to the medical inspection of school-children. The Act is purely a consolidating one, and contains no amendments, and it is to be hoped that it will soon be followed by similar Acts dealing with the law of public health. If the Ministry of Health wishes to regain some of its lost popularity, here are the means ready to hand.

---

### THE LEAGUE OF MERCY.

At the twenty-second annual meeting of the League of Mercy, which was held at St. James's Palace, Princess Alice Countess of Athlone, who presided, read a letter from the King in which His Majesty congratulated the League on having collected and distributed, since its foundation by King Edward in 1899, a sum of no less than £360,000. The Princess said that she did not believe the League had ever been going stronger. They had always endeavoured to work on friendly terms with all other societies which were also working to help the hospitals; they were careful not to clash with the special appeals of individual hospitals and to work side by side with central funds. The Executive Committee was devoting close attention to all the questions arising out of the various schemes suggested by which certain hospital and other benefits were secured to persons contributing small weekly sums. She mentioned that a new Domestic Workers' Association was successfully extending the work of the League among persons in domestic service.

Lord Cave, in the course of his speech, said that by far the greatest deficiency in hospital funds arose in those cases where medical schools were attached. He thought that in future a grant in aid to teach should be made. He expressed the hope that the Chancellor of the Exchequer would consider his Committee's recommendation that gifts intended for public service should no longer be subject to a reduction of 10 per cent. before they reached the treasurers of the hospitals. He hoped that the great bulk of friendly and provident societies would follow the example of those which had already consented to make grants to hospitals. As to mass contributions, it was a striking fact that in some places useful funds had been provided by persons of small means. While he did not wish to commend one scheme in preference to another, he felt strongly that here was a source of income which should be considered by hospital managers. It had been suggested that employers of labour should be asked to set aside a percentage of their profits as contributions to hospitals; if that system could be started a considerable amount might be secured, but after all, he believed that the future of the hospitals would continue to depend to a very large extent on voluntary contributions. He hoped that the League of Mercy, which contributed yearly to King Edward's Hospital Fund, would extend its work and help the hospitals outside the London area until there was no part of England, and possibly Scotland, without a branch of the League.

The Hon. Treasurer stated that the amount collected during the year was £34,400.

